# School health services in the Southern District of Israel—between privatization and nationalization

**DOI:** 10.1186/s13584-024-00647-3

**Published:** 2024-10-07

**Authors:** Tzion Dadon, Ya‘akov M. Bayer, Anat Rosenthal, Michael Gdalevich

**Affiliations:** 1 Ben-Gurion University of the Negev, Ben Gurion, Blvd 1, Beer-Sheva, Israel; 2 Ministry of Health, Beer-Sheva, Israel

**Keywords:** Public health, Pupil health, Vaccination coverage, Screening tests, Health education

## Abstract

**Background:**

For decades, Israel’s economic policy has favored either outsourcing or privatization of public services, including healthcare, generating an ongoing and prolonged debate of this approach. In 1997 school health services (SHS) for elementary and middle school pupils was outsourced to a sub-contractor firm, reducing budget, but also standards, for nurses and physicians. Consequently, the service has dwindled and was focused more and more on vaccinations. Between 2007 and 2012, under full private contractor delivery, SHS quality diminished substantially, leading to a significant decline in vaccination coverage in the Southern District. In 2012, a decision was made to return SHS to state control.

**Methods:**

This study analyzes the delivery parameters of SHS between the period when the service was operated by a private contractor from 2011to 2/2012, and the subsequent provision of the service directly by Ministry of Health (MoH) between 3/2012 and 2013. We compared the rates of vaccination coverage, screening tests and health education programs.

**Results:**

A statistically significant increase in SHS delivery for vaccinations and screening was observed in the Southern District of MoH after the transfer of service from contractor. The increase was variable in different population subgroups, and especially notable in the Bedouin schools of the District, where the MMRV vaccination rose from 19.3% to 96.8%. However, a substantial and significant reduction in health education activities was also noted, overall from 24.9% to 5.0%.

**Conclusions:**

The findings suggest that substantial benefits can be derived from direct provision of SHS by MoH and its regional offices, especially in the areas of reduced accessibility and lower socio-economic status. The case study of SHS in the Southern District of Israel can serve as an important example highlighting the impacts of privatization vs nationalization, with potential implications in other fields. These insights should be integral to future discussions of healthcare service provision.

## Introduction

The privatization versus nationalization of health services has generated abundant debate in health systems worldwide. While many see privatization as the answer to growing demands and costs, others see it as a threat to the professional integrity of the system. In the context of these debates, this study aims to explore the provision of school health services in the Southern district of Israel, with a focus on the transitions from privatized services to the MoH. By examining the outcomes of these transitions, this study seeks to evaluate the effectiveness of health services delivery through both private and public mechanisms and contribute to the ongoing debates on the privatization of health services in Israel.

## Background

### Privatization and nationalization of health services

The rapid growth in medical technologies, increased demand for healthcare services, and population aging have led to a healthcare crisis. This has influenced the funding of the healthcare system and forced many countries to privatize or increase private funding within public systems. The funding landscape in the healthcare system has changed, and citizens who previously benefited from extensive public funding have had to participate privately in healthcare costs. These changes have also been reflected in the ownership and organizational structure of healthcare systems in the Western world. Consequently, many Western countries provide their citizens with universal healthcare coverage that offers a basic basket of services, alongside the option to secure additional extended coverage through private insurance. Sources of healthcare funding may come from general or specific government taxes, as well as from citizens' contributions through direct payments and participation in premiums [1].

The privatization of healthcare systems worldwide has generated significant interest, and many researchers in the field believe that privatization "commercializes" medical services and impacts healthcare negatively [2].In Italy, after four decades of experience with healthcare privatization similar to the reforms enacted or proposed in other countries, it has been concluded that significant cuts in funding for national healthcare services and the expansion of services in the private sector have hampered the ability to deal with healthcare emergencies. This was evident at the beginning of COVID-19)pandemic, which severely affected Italy and forced extreme measures to bolster resources in public hospitals [3].

Over the past few decades, countries around the world have often embraced a liberal market economic strategy, favoring privatization and outsourcing in circumstances where there are no significant market failures and such actions are deemed economically viable by governmental bodies. This approach frequently entails the delegation of tasks to private entities rather than a complete transfer of ownership, with the allocation of public funds remaining intact. This setup aims to harness the efficiency and expertise of the private sector while ensuring accountability and oversight from the public sector. However, it is worth noting that while this approach is often perceived as inherently correct, there are instances where it fails to prove its economic efficiency and negatively impacts service quality.

Privatization of health services goes beyond the simple transfer of ownership and may take different forms and shapes [4]. In fact, privatization in health services does not necessarily follow to strict definition of transfer of ownership and responsibility from the state to private enterprise, but may also include policies whereby “tasks are taken over by the private sector, but responsibility remains with the public sector” [5]. In this context, notable forms of health service privatization worldwide include outsourcing, out-of-pocket pay for services and limitations put on public provision to encourage private activity [6].

In Israel, like many other countries worldwide, the economy was characterized largely by concentration and the provision of services through the public sector until the late 1970s. In the past decades, Israeli society has transitioned from an economy characterized by high government involvement to a more liberal market economy, reducing the public sector and privatizing various services [7]. Many services that the government previously provided for many years were transferred to private hands [8], including public goods, public services, national infrastructures, and various other fields of service. Within this framework, some healthcare and welfare services were also privatized, either fully or through outsourcing, subcontracting, regulated competition, and granting licenses [9]. In practice, government institutions have narrowed their activities to regulatory, supervisory, and enforcement activities aimed at directing and supervising activities in cases where it is essential, such as public goods, public services, and critical infrastructure [10].

In the past two decades, the Israeli government has adopted a stance that emphasizes reducing government involvement in the economy while increasing the role of private entities. This approach aims to promote competition, enhance efficiency, reduce government expenditures, and encourage investments, ultimately leading to a strong and healthy economy that benefits everyone. In this way, the government can cut its spending, and the public can enjoy cheaper and better services. While this approach has proven itself in many countries and across various sectors of the economy, it should be subject to deep scrutiny and discussion, especially in the fields of healthcare and welfare. The limited debate on privatization resulted in outsourcing and privatization processes that have shown themselves to be prone to shortcomings, particularly in terms of supervision and control, which, in many cases, have resulted in a significant decline in the quality of government-provided services in these areas [11].

The healthcare system in Israel is primarily a public healthcare system built on the foundation of the healthcare system that operated in the country during the British Mandate period [12]. In 1995, the National Health Insurance Law was enacted, making health insurance mandatory for all residents of the country, with each individual being insured by one of the four existing health maintenance organizations (HMOs) of their choice. Except for mental health services, geriatrics, and preventive medicine which remained under the jurisdiction of the MoH, all other healthcare services were transferred to the HMOs [13]. The Ministry of Finance opposed the National Health Insurance Law from the day it was legislated, based on two principles: first, to minimize public spending as much as possible, and second, to prioritize private services over government services whenever possible. Consequently, a decision was made to privatize preventive healthcare services, including student health and mother and child [*tipat halav* in Hebrew] programs, which remained under the Ministry of Health's purview [14]. The service baskets provided under the National Health Insurance Law suffer from underfunding that does not consistently take into account the growth in the population, population aging, increased healthcare expenditures, and technological advancements occurring each year. This has resulted in a gap between the desired funding level and the actual funding provided. As a result, hospitals and HMOs began operating on a "business model", offering supplementary insurance and private services [15].

Similarly, SHS which were provided by the state, were privatized following the National Health Insurance Law of 1995 and transferred to the management of private companies. These services were partially returned to the management of the Ministry of Health in the Southern, Ashkelon, and Northern districts, while in other districts, the service remains privatized.

## School health services in Israel

Health services in schools were established as early as the 1920s (under the British Mandate of Palestine-Eretz Israel), initiated by the Hadassah organization.^1^ In each school, "nurses' rooms" operated as makeshift clinics, providing first aid services to children, conducting periodic check-ups, identifying developmental issues, and referring children for specialized treatments [16].

Following the establishment of the state of Israel in 1948, the school clinic model was expanded under the guidance of the Ministry of Health as part of a program aimed at promoting public health. Each school had a clinic managed by a nurse employed by the MoH, who provided vaccinations, health education outreach, primary medical care, and home visits to address pupils' unique health needs, thereby instilling hygiene practices among pupils and reducing morbidity rates, particularly for infectious diseases [17].

Over time, the responsibility for providing health services in schools shifted to municipalities, either directly or through private companies, as well as the MoH and the General Health Insurance Fund. The municipalities were allowed to charge parents a fee for these services in accordance with the Compulsory Education Law (1949).

However, with the enactment of the National Health Insurance Law in 1995, the responsibility for providing health services to pupils was transferred to the health funds (non-profit HMOs) or their service providers. The law also specified that these services would be funded by the state treasury, absolving municipalities of their responsibility to provide health services and charge fees for them. In practice, since 1997, the MoH has outsourced the service to the Health Association, which acts as a personnel contractor, but the overall responsibility remains with the Ministry [18].

In the mid-2000s, the SHS budget was reduced due to an ongoing dispute between the Treasury and the MoH regarding the desired nature of the service. According to the MoH approach, health services for schoolchildren should be comprehensive and derived from a broad view of needs, not limited to performing a set of tasks. According to this approach, public health nurses would be an integral part of the school team and actively involved in the school and community. In contrary, according to the Ministry of Finance, health services for pupils are a collection of defined tasks (vaccinations and examinations) that should be performed at school, and there is no need for a permanent presence of a nurse at the school for the proper provision of these services. Therefore, the service is primarily evaluated based on the coverage of vaccinations and screening tests [19].

In 2006, under pressure from the Treasury, it was decided that SHS would be provided by the Public Health Association (a private contractor), rather than as a human resources contractor. In fact, this is a process of privatizing the service, in which the government removes the service from its hands and allows the Public Health Association to provide it without a tender for a period of 3 years. In addition, the service was outsourced so that first aid services for schools are provided by an external entity, initially by Magen David Adom (MDA)^2^ and later by NATALI.^3^

In July 2009, the government published a tender for service provision. NATALI won this tender in addition to the tender for providing first aid services to schools. The entities that lost in the tender filed a lawsuit against NATALI’S win, and the entire service went through a lengthy process of legal disputes, adding uncertainty to the service and the employed nurses. NATALI struggled to properly operate the service, and serious problems were discovered in the working conditions of the nurses in the service [20]. In mid-2012, the Supreme Court issued a ruling in an appeal filed by the Israeli Medical Association, sharply criticizing the government’s handling of health services for pupils. The Supreme Court determined that the state had failed both in managing the entire service and in inadequate supervision, and called on the state to consider nationalizing the service [21]. Control of of these services returned to the MoH.

Currently, the question of the provision of public health services is being raised again. Specifically, whether the Mother-and-Child clinics, as well as the SHS, should be provided directly by the state or through external contractor agencies. We believe that the current analysis of our data sheds some light towards the discussion.

## Methods

This study examines the outcomes of SHS in Israel during periods when it was operated through privatized and nationalized delivery mechanisms.

The research includes data on the health services provided to pupils from first grade to the ninth grade in 319 schools scattered across 29 municipalities within the jurisdiction of the Southern District of the MoH, as recorded in the Israel's national vaccination registry during the years 2011–2013. The data includes information on vaccination coverage, screening tests (vision and hearing), and health education carried out in schools in the Southern District during the period of operation of the SHS by a private contractor (NATALI, 01.09.2010 to 29.02.2012) compared to the period of operation by the MoH (public health services, 01.03.2012–21.08.2013).

The independent variables in the study were categorized according to service providers (NATALI and the MoH). Dependent variables were data on health promotion activities for pupils in each school. The variables were classified identically for all health promotion activities for pupils (vaccinations, surveys, and health education) by the service provider (NATALI/MoH), schools by sector (Jewish, permanent settlement in Bedouin localities, and dispersed Bedouin), type of authority (municipality, local council, and regional council), type of educational supervision (secular, religious, and ultra-Orthodox), type of education (regular and special), and ethnic group. Univariate analysis was performed using statistical tests to examine differences in policy outcomes (vaccinations, survey tests, and health education) when the differentiating variable was the mode of operation (private and public). This section uses cases where homogeneous groups with similar characteristics are involved.

A comparison was made between service providers (NATALI—private and MoH—public) using a Chi-square test (= 3.84(df(1,0.05,) with statistical significance defined as p < 0.05, and calculation of the percentage difference in coverage between service providers (Δ (public–private) and the confidence interval (CI) was conducted.

## Settings

The population of the Southern District in Israel is diverse and comprised of various ethnic groups and different settlement patterns. The population includes Jews and Muslims. Most of the Jewish population resides in cities, towns, and rural settlements with a moderate socio-economic status and is defined at a social-economic level^4^ as medium to low (levels 3–5). Another subpopulation in the Southern District is the Haredi^5^ community, which accounted for 12.5% of the total population of the country in 2020. The natural growth rate of the Haredi Jewish population is 4% per year, compared to 1% in the non-Haredi Jewish sector. The majority of Haredim work part-time jobs, reflecting their low monthly income compared to the general sector [22].

The Arab population is composed of residents who live in towns and those who reside in dispersed rural settlements. Many families in these areas live in buildings that were constructed illegally and lack appropriate infrastructure. Some of these towns are not recognized by the central government, and as a result, they do not receive the full infrastructure provided by the government to recognized towns. Many of these communities exist in difficult conditions without suitable infrastructure. Almost all the Arab population in the South identifies as Bedouin, a Muslim population originally rooted in a nomadic culture. This population consists of several tribes scattered across different areas in southern Israel and is defined at the lowest socio-economic level (level 1).

The growth rate of the Bedouin population is exceptionally high. Between the years 2000–2019, the Arab population in the Be'er Sheva region grew by 144%, which is nearly 2.5 times its size during that period. In contrast, the Jewish (and other) population in the Be'er Sheva region grew by 29% [23].

## Results

### Routine vaccinations—a comparison between the NATALI company (private) and the MoH (public)

According to the routine vaccination program of the MoH the mandatory school vaccination schedule includes: In first grade—MMRV vaccine, in second grade—Tdap-ipv vaccine, and in eighth grade—Tdap vaccine [24].

As seen in Table 1, when comparing vaccination coverage rates between the MoH and NATALI, it was found that during the MoH period, there was a significant increase in vaccination coverage and a clear statistical difference (p < 0.001). For MMRV vaccination, there was an increase of 42.8%, for Tdap-ipv vaccination an increase of 39.8%, and for Tdap vaccination an increase of 46.8%. This trend was also observed when analyzing the population by demographic groups.

**Table 1:** Vaccination Coverage in first, second and eighth grades

	**Private (NATALI)**		**Public (Ministry of Health)**		**Comparison**		
Type of vaccine	N Number of pupils	% vaccinated (n- Number of pupils vaccinated)	N Number of pupils	% vaccinated (n- Number of pupils vaccinated)	Δ (Public–Private) (CI)	df	*x*^2^
MMRV	28,462	**51.83%**(14,752)	23,069	**94.62%**(21,829)	**42.8%**(42.3%-43.6%)	1	11,330.2***
Tdap-ipv	26,441	**53.38%**(14,115)	23,314	**92.57%**(21,582)	**39.8%**(38.3%-40.1%)	1	9386.0***
Tdap	21,545	**39.42%**(8,493)	19,360	**87.24%**(16,696)	**46.8%**(45.8%-47.9%)	1	9447.6***

^***^p < .001

In accordance with the details provided in Table 2, a significant increase in vaccination coverage was found by demographic sector during the period when the service was provided by the MoH compared to the period when it was provided by NATALI. For the MMRV vaccine, among the Jewish sector there was an increase of 25.3%, among Bedouin permanent settlements an increase of 55.4%, and among Bedouin scattered settlements an increase of 76.9%. For the Tdap-ipv vaccine, among the Jewish sector there was an increase of 24.2%, among Bedouin permanent settlements an increase of 54.6%, and among Bedouin scattered settlements an increase of 66.7%. And for the Tdap vaccine, among the Jewish sector there was an increase of 36.3%, among Bedouin permanent settlements an increase of 66.2%, and among Bedouin scattered settlements an increase of 71.1%.

**Table 2:** Vaccination coverage in first, second and eighth grades by demographic sector

		**Private (NATALI)**		**Public (Ministry of Health)**		**Comparison**		
Type of vaccine	Sector	N Number of pupils	% vaccinated (n- Number of pupils vaccinated)	N Number of pupils	% vaccinated (n- Number of pupils vaccinated)	Δ (Public–Private) (CI)	df	*x*^2^
MMRV	Jewish	14,713	**69.79%**(10,268)	10,989	**95.07%**(10,447)	**25.3%**(24.2%-26.4%)	1	2574.9***
	Bedouin—permanent settlement	9,607	**37.93%**(3,644)	8,393	**93.36%**(7,836)	**55.4%**(54.0%-56.9%)	1	5957.9***
	Bedouin – Diaspora	4,142	**19.31**(800) %	3,687	**%96.18**(3,546)	**76.9%**(75.1%-78.7%)	1	4565.5***
Tdap-ipv	Jewish	14,660	**68.73%**(10,076)	11,110	**%92.93**(10,325)	**24.2%**(23.0%-25.4%)	1	2244.6***
	Bedouin—permanent settlement	8,438	**%38.54**(3,252)	8,532	**%93.10**(7,943)	**54.6%**(53.0%-56.1%)	1	5624.5***
	Bedouin – Diaspora	3,343	**23.54%**(787)	3,672	**%90.25**(3,314)	**66.7%**(64.5%-69.0%)	1	3206.6***
Tdap	Jewish	13,192	**52.73%**(6,956)	10,276	**89.05%**(9,151)	**36.3%**(35.0%-37.7%)	1	3540.2***
	Bedouin—permanent settlement	5,862	**% 21.43**(1,256)	6,357	**%87.60**(5,569)	**66.2%**(64.4%-67.9%)	1	5416.8***
	Bedouin – Diaspora	2,491	**%11.28**(281)	2,727	**%82.36**(2.246)	**71.1%**(68.6%-73.6%)	1	2633.6***

^***^p < .001

### Screening tests and health education—Comparison between NATALI (private) and the MoH (public)

The growth, vision, and hearing Screening tests are conducted by health nurses for pupils in schools, with the aim of early identification, to the extent possible, of pupils with health issues, in order to provide them with effective treatment to improve their functioning in studies and integration into society [25].

As depicted in Table 3, we found an increase in the coverage of screening tests during the period of the MoH compared to the period when the service was operated by NATALI. In growth tests in the first class and the seventh class, there was an increase of 8.4% and 13.1% respectively. In vision tests in the first class and the eighth class, there was an increase of 11.4% and 1.6% respectively. And in hearing tests in class A, there was an increase in coverage of 32.9% during the MoH period.

**Table 3:** Coverage of survey tests in first, seventh, and eighth grades

		**Private (NATALI)**		**Public (MoH)**		**Comparison**		
**type of test**	**Classroom**	N Number of pupils	% vaccinated (n- Number of pupils tested)	N Number of pupils	% vaccinated (n- Number of pupils tested)	Δ (Public–Private) (CI)	df	*x*^2^
**Growth test**	first	28,309	**%80.98**(22,927)	17,272	**89.36%**(15,435)	**08.4%**(07.5%-09.2%)	1	564.5***
	Seventh	21,409	**%64.18**(13,742)	17,792	**77.27%**(13,748)	**13.1%**(11.9%-14.3%)	1	793.8***
**Eye test**	First	27,838	**67.63%**(18,827)	19,315	**79.07%**(15,272)	**11.4%**(10.4%-12.5%)	1	745.1***
	Eighth	19,333	**6%50.6**(9,795)	21,496	**52.29%**(11,240)	**01.6%**(0.30%-02.9%)	1	10.7
**Hearing Test**	First	27,635	**%38.93**(10,757)	24,097	**%71.85**(17,314)	**32.9%**(31.9%-34.0%)	1	5622.8***

^***^p < .001

### Health education- Comparision between NATALI (Private) and the Ministry of health (Public)

Health activity for pupils in schools is at least one activity in each class, and as part of the training, topics from the health education program can be incorporated into the instructions.

According to the information presented in Table 4, when comparing the percentage of health education activity in classes1-9, a decrease of 19% was found during the period of the MoH compared to the period when the service was operated by NATALI.

**Table 4:** Coverage of health education activity in the first class to the ninth class

	**Private (NATALI)**		**Public (MoH)**		**Comparison**		
Type of Activity	N Number of classes	% Instruction (n- Number of classes that have been trained)	N Number of classes	% Instruction (n- Number of classes that have been trained)	Δ (Public–Private) (CI)	df	*x*^2^
Health Education Grades 1–9	9,061	**24.92%**(2,258)	7,842	**04.99%**(391)	**-19.9%**(-21.3%–18.6%)	1	1263.9***

^***^p < .001

## Discussion

The findings indicate that vaccination rates and screening tests coverage rates of the target population improved significantly when administered by the MoH compared to the NATALI. Health education coverage during the research period was found to be higher during the MoH period compared to the NATALI. The results of the study show that vaccination coverage while under MoH was approximately 90%-95% thus fitting the requirements of herd immunity in ethnically mixed areas [26], as in the case of this examination, while under NATALI, the coverage was approximately 50%, and in the Bedouin sector it was approximately 30%. Survey testing coverage during the MoH period was approximately 74%, with an average of 14% in the Jewish and 20% in the Bedouin population, compared to the corresponding period of NATALI. Health education coverage was low in both periods. During the MoH period, there was a decrease of approximately 20% in health education coverage compared to the NATALI period.

The literature exposes a complex landscape concerning healthcare in Europe, offering substantiation of a noticeable trend towards heightened privatization. Public healthcare spending has halted its growth since the 1980s, and in select nations, there has been a substantial increase in the involvement of the private sector [27]. In the study by Katan & Lowenstein [28], the implementation of social services privatization policy is one of the indicators of recent changes in the nature of the welfare state in many Western countries, including Israel. One of the implications of such policy is that many services that governments and local authorities are obligated by law or interested in providing to various sectors of society are actually provided by private organizations, including voluntary organizations and commercial for-profit entities. The process of privatization takes on various forms in these countries and in different scopes in all aspects of social services, including health, income security, education, housing, employment, and personal welfare services. Gronau [29] argued that in recent years, the Israeli government has been transferring a growing portion of social services, including health services, to private providers, and this process harms those service recipients, who are often among the weakest and most vulnerable members of society. In his study, he explains the importance of public management in this sector. Competition inherently fosters economic efficiency when consumers can readily identify and select the most suitable provider. However, often service consumers are not able to assess the quality of the services they receive, and there is a lack of transparency in the quality of services provided by private organizations, which may affect service recipients’ ability to access appropriate and high-quality services. This raises questions about accountability, regulation, and oversight in the privatization of social services, including health services.

This situation of competition for welfare and health services may harm vulnerable populations if it is not regulated by the government. These claims are supported by the findings of this study, as the privatization of SHS in the Southern district was detrimental to the service and its delivery, especially towards vulnerable subgroups in the south, due to the independent management of the service by private companies. In addition, the supervision of the service in the southern district during the privatization period was carried out only from a professional perspective by supervisory nurses for pupils’ health checks in schools, and not on the level of comprehensive management of the service. This situation has led to independent management by the companies without supervision and control. When the service was transferred to direct operation by the Southern District Health Bureau, the service delivery significantly improved and met the required standards, with a coverage rate of 90%-95%.

Obviously, it is important to consider the impact of privatization of health services on vulnerable populations and the need for proper governmental regulation and oversight to ensure high-quality services and equitable access-for-all healthcare. For decades, Israel’s economic policy has generally leaned towards a liberal market approach, promoting privatization and outsourcing, where there are no market failures and while economically justified according to fiscal authorities. This approach can manifest as outsourcing rather than outright transfer of ownership, depending on the specific case and circumstances. An expression of this policy can be found in the case examined in our research, where public funding is maintained while execution is carried out by private entities. Our research examines the outsourcing of school health services (SHS) for elementary and middle school pupils in 1997, which aimed to reduce costs but inadvertently lowered standards for nurses and physicians. Consequently, the service was significantly reduced and focused primarily on vaccinations. Between 2007 and 2012, when SHS was fully managed by private contractors, there was a notable decline in service quality and a significant drop in vaccination coverage in the Southern District. As a result, in 2012, a decision was made to return SHS to state control.

While the findings of this study support a critical review of privatization policies, some limitations exist. It was not possible to examine the cost–benefit ratios of the various operators within SHS. Additional research is needed in order to examine the financial aspect, which has significant impact in determining the supply mechanism. Furthermore, we recommend expanding the study to other districts to explore the implication of privatization of pupil health services country-wide.

## Conclusions

Based on the research findings, it appears that, in specific situations, government provision of healthcare services could be a more effective approach than the prevalent economic theory and trend of privatization in the Western world. The research presents an example where efforts to establish such a mechanism through privatization and competitive management via tender have been unsuccessful, and only government provision of the service has yielded satisfactory outcomes. These findings should be considered when further privatization process are deliberated in the future.

## Data Availability

The data supporting the findings presented in this article are available through a designated repository. Link to datasets: https://osf.io/prhzs/files/osfstorage/655cdc50f6ce3c3b81504f5c

## Abbreviation

**SHS**: School health services

## Footnotes

1 Hadassah, The Women's Zionist Organization of America is an American Jewish volunteer women's organization. Founded in 1912 by Henrietta Szold.

2 Magen David Adom in Israel is the national rescue organization of the State of Israel since its inception for pre-hospital emergency medical services and blood service. Magen David Adom serves as the Israeli Red Cross organization, it is a full member of the International Red Cross/Red Crescent organization.

3 NATALI- is one of the oldest, largest and most experienced private medical services companies in Israel that has been operating for over 30 years.

4 Socioeconomic -Level—The Central Bureau of Statistics formulated an index to characterize the local authorities in Israel according to the social level -economic of the population. The municipalities and local councils were classified according to the index values ​​into ten levels, where level 1 indicates the lowest socio-economic level and level 10 indicates the socio-economic level.

5 Groups within Orthodox Judaism distinguished by their rigorous observance of halakha (Jewish law) and customs.
